# Novelty to necessity: large language models in clinical practice—a call to action for physicians

**DOI:** 10.1007/s11739-026-04393-z

**Published:** 2026-05-17

**Authors:** Vittorio Beltrani, Olivier Giannini, Omar F. AbouEzzeddine, Martin R. Gruebler, Federico Mastroleo, Gregorio Tersalvi

**Affiliations:** 1 Department of Cardiology, Istituto Cardiocentro Ticino, Lugano, Switzerland; 2 Department of Internal Medicine, Ente Ospedaliero Cantonale, Bellinzona, Switzerland; 3 Faculty of Biomedical Sciences, Università Della Svizzera Italiana, Lugano, Switzerland; 4 Department of Cardiovascular Diseases, Mayo Clinic, Rochester, MN USA; 5 Department of Cardiology, University Hospital Neustadt, Danube Private University, Wiener Neustadt, Austria; 6 Department of Internal Medicine, Medical University of Graz, Graz, Austria; 7 Medical Faculty, Sigmund Freud University, Vienna, Austria; 8 Department of Radiation Oncology, Mayo Clinic, Rochester, MN USA; 9 Division of Radiation Oncology, European Institute of Oncology, IEO European Institute of Oncology IRCCS, Milan, Italy; 10 Department of Oncology and Hemato-Oncology, University of Milan, Milan, Italy; 11 Ospedale Regionale Di Mendrisio, Via Alfonso Turconi 23, 6850 Mendrisio, Switzerland

**Keywords:** large language models, clinical practice, artificial intelligence, physician accountability, calibrated trust, healthcare governance

## Abstract

Large language models (LLMs) represent a rapidly advancing subset of artificial intelligence with significant potential to augment clinical practice. These tools can assist in literature synthesis, documentation, clinical reasoning, and patient communication. Despite demonstrated capabilities, adoption in healthcare remains limited due to barriers such as limited AI literacy, trust concerns, integration challenges, and generational disparities in digital proficiency. This review critically examines the role of LLMs as cognitive adjuncts in medicine, emphasizing the need for calibrated trust, human oversight, structured implementation strategies, and robust regulatory governance. Evidence suggests that stepwise integration of LLMs, beginning with low-risk tasks, may enhance physician efficiency, reduce cognitive burden, and may support high-quality care. Key limitations, including hallucinations, data currency, environmental impact, and data security, have to be addressed, alongside the need for continuous model refinement. Rigorous clinical trials are needed to establish the efficacy of LLMs. This includes the recognition that LLMs trained on vast web data may contain personal health information, creating a significant privacy risk. Responsible adoption of LLMs is essential to meet the demands of modern medicine while preserving clinical accountability and patient-centered care.

## Introduction

In 2022, a tool became publicly available that could pass medical licensing examinations, summarize clinical guidelines, and draft discharge letters in seconds [1]. Within months, it had entered the clinical workspace, often without institutional guidance, validated protocols, or physician training [2]. The rapid and largely unstructured diffusion of large language models (LLMs) into healthcare practice represents both an extraordinary opportunity and an underappreciated risk and it is this tension that the present narrative review seeks to address.

AI represents a broad field focused on developing systems that perform tasks typically requiring human intelligence, such as learning, reasoning, and language understanding. LLMs, including tools such as ChatGPT, DeepSeek, LLaMA, and Claude, constitute a specialized category of AI technology that can interpret queries, synthesize medical literature, and generate clinical documentation [3]. The phrase “generative AI” became popular in the early-to-mid 2020s, particularly after the public release of ChatGPT in November 2022, and refers broadly to AI systems capable of generating text, images, audio, and other media [4]. For the purpose of this review, we will use the term LLM to encompass these text-based cognitive tools. To ensure conceptual clarity for clinical readers, we have included a dedicated glossary (Table 1) defining key terms including AI, LLM, Generative AI, Hallucination, and Retrieval-Augmented Generation (RAG), as these concepts are frequently used interchangeably in the literature despite meaningful technical distinctions.

**Table 1:** Glossary of key artificial intelligence and large language model terminology for clinicians

Term	Definition	Clinical relevance/example
Artificial intelligence (AI)	The broad field of computer science dedicated to creating systems capable of performing tasks that typically require human intelligence (e.g., reasoning, learning)	The "umbrella" term covering everything from simple ECG algorithms to complex diagnostic tools
Generative AI (GenAI)	A subset of AI capable of creating new content (text, images, audio, or code) rather than just classifying or predicting existing data	Used to draft patient letters, generate educational illustrations, or summarize medical records
Large language model (LLM)	A type of GenAI trained on vast datasets of text to understand, summarize, and generate human-like language based on statistical probabilities	The engine behind tools like ChatGPT, Claude, or DeepSeek used for literature synthesis
Augmented intelligence	A conceptual framework where AI is designed to enhance human intelligence and decision-making rather than replace it	The physician remains the "pilot," using AI as a cognitive amplifier to manage data overload
Retrieval-augmented generation (RAG)	A technique that links an LLM to a specific, trusted external database (e.g., PubMed or hospital guidelines) to ensure the output is grounded in facts	Crucial for reducing "hallucinations" by forcing the model to cite specific clinical evidence
Hallucination	A phenomenon where an LLM generates factually incorrect or fabricated information that sounds highly plausible and confident	A primary safety risk; necessitates the “TRUST Safety Loop” for clinician verification
Vibe coding	A contemporary term for building functional software or "apps" using natural language instructions (LLMs) instead of traditional programming languages	Allows clinicians to create personalized workflow tools or calculators without needing to learn Python or Java
PICO framework	A structured method for framing clinical questions: Population, Intervention, Comparison, Outcome	Essential for optimizing “prompts” (instructions) given to an LLM to get high-quality clinical answers

The integration of LLMs into healthcare is increasingly being considered as a potential response to growing informational and administrative complexity. As the volume of medical information continues to grow rapidly, traditional approaches to knowledge acquisition and decision-making are becoming increasingly inadequate for maintaining optimal patient care standards [5]. In this framework, LLMs should be viewed not as autonomous replacements for physicians, but as “Augmented Intelligence” partners engineered to reinforce and refine the delivery of modern healthcare. Just as the stethoscope extended the physician’s auditory range, LLMs serve as cognitive amplifiers, allowing clinicians to navigate the otherwise unmanageable landscape of contemporary medical data.

Yet adoption remains limited and inconsistent. Many physicians who initially explore LLMs abandon them after brief use, perceiving them as adding complexity rather than reducing workload. This “review burden”, the cognitive effort required to verify LLM outputs for accuracy, often offsets perceived efficiency gains [6]. This perception stems not only from inherent technical limitations but also from a significant knowledge-practice gap, where high LLM performance on medical examinations does not necessarily translate to safe clinical integration [7]. The disconnect lies in treating LLMs as plug-and-play software rather than complex instruments requiring a distinct set of learned competencies. Reported barriers to adoption include the scarcity of prospective randomized controlled trials establishing clinical efficacy, limited AI literacy regarding generative architectures, concerns about explainability, and the absence of structured institutional frameworks to guide safe implementation [8, 9].

This narrative review, therefore, aims to bridge the gap between technological novelty and clinical necessity by: (1) analyzing current barriers to LLM adoption, with particular attention to the generational digital divide and the trust deficit; (2) proposing a structured framework, the Practical Framework and TRUST Safety Loop, to guide safe, stepwise integration into daily practice; and (3) critically examining the ethical, environmental, and regulatory boundaries that must define this new era of augmented medical practice.

## The implementation challenge: skill acquisition and the generational gap

The primary challenge in adopting LLMs is fundamentally socio-technical, involving a steep learning curve within a rapidly shifting professional landscape. This transition can be illustrated by the metaphor of walking versus driving a manual car. Walking, like traditional clinical workflows, feels natural, intuitive, and provides a sense of control and familiarity. In contrast, learning to drive is initially awkward, requiring the frustrating coordination of clutch, gears, and accelerator. Yet once mastered, the vehicle becomes an extension of the driver, enabling far greater speed and efficiency. To dismiss LLMs after a brief unsuccessful trial is therefore akin to abandoning a car after stalling it once: a missed opportunity to unlock unprecedented efficiency and access to knowledge.

This learning curve is further complicated by a significant generational digital divide. Digital natives often demonstrate intuitive adaptability to LLM interfaces, integrating them rapidly into daily practice. Conversely, senior clinicians, who frequently hold the greatest clinical responsibility and institutional oversight roles, may differ widely in their prior experience with emerging digital tools and therefore more cautiously demand evidence-based validation and formal Health Technology Assessment before deployment [8]. Both positions are legitimate, but narrowing this divide will require structured, competency-based training in addition to access to new tools, as emphasized in the 2025 DECODE international consensus framework for digital health in medical education [10, 11].

LLMs offer multiple functionalities, from summarizing research papers to supporting clinical reasoning and deep literature exploration. Each tool has its strengths and these models are evolving rapidly, such that what seems limited today may become indispensable tomorrow [7, 12, 13]. Yet the evidence suggests that high LLM performance on medical examinations does not automatically translate into safe or effective clinical integration [14]. The disconnect lies in treating LLMs as plug-and-play software rather than complex instruments requiring a distinct set of learned competencies. The key determinant of sustainable adoption is therefore not familiarity alone, but the development of structured institutional competencies that allow clinicians of all generations to use these tools safely and responsibly [8, 11, 15].

## Calibrating trust and global governance

Trust remains the fundamental barrier to AI adoption in healthcare. Beyond simple acceptance, the focus has shifted toward “calibrated trust”: a balanced state where clinicians neither over-rely on AI nor dismiss it prematurely. Sagona et al. emphasize that trust in AI-assisted health systems depends not only on clinician confidence in the system, but also on system design that supports appropriate human oversight [16]. To formalize this nexus, a global consensus is emerging. The transition from novelty to necessity requires moving beyond enthusiasm toward standardized reporting and methodological quality to ensure patient safety [17, 18].

Current institutional perspectives reflect this shift:

- North America: The American Medical Association (AMA) emphasizes that AI should “augment rather than replace” clinical judgment [19], while the American College of Physicians (ACP) advises against using anthropomorphic language such as “thinking” to prevent unwarranted over-reliance [20].
- Europe: The Italian Society of Internal Medicine (SIMI) advocates for a “human warranty” principle, ensuring that AI outputs are always validated by a human expert [21]. Similarly, the UK’s NICE stresses the necessity of outcome-blinded assessments to prevent bias in evidence generation [22]. The EU’s Trustworthy AI guidelines establish seven foundational principles: human oversight, technical robustness and safety, privacy and data governance, transparency, diversity and non-discrimination, societal and environmental well-being, and accountability [23]. These pillars are increasingly instrumental in defining the classification and governance of LLMs developed for clinical applications.

## The evolution of medical responsibility: a shifting paradigm

Central to this debate is the evolving principle of medical responsibility. A prevailing proposal, often illustrated by the automobile metaphor, suggests that the “steering wheel” of clinical decision-making should remain firmly under the physician’s control to ensure patient safety and preserve the humanistic essence of the patient-physician relationship [24]. However, this model is increasingly a subject of critical re-examination. It is no longer taken for granted that the physician will, or should, always be the final decision-maker in an era of high-autonomy AI.

Some scholars argue that, as AI systems demonstrate superior performance in specific diagnostic or predictive tasks, rigid adherence to human-led oversight might even be counterproductive to patient safety. This shift necessitates a clarification of accountability frameworks: the medical community is now debating whether professional responsibility should remain centered on the individual clinician or be redistributed across a shared architecture involving technology developers and institutional governance structures [25, 26]. A significant concern in this transition is the risk of physicians becoming "liability sinks", held legally and ethically accountable for the outputs of “black-box” algorithms that they can neither fully interpret nor effectively override in real-time [25]. Thus, the transition requires not just technical trust, but a fundamental rethink of who, or what, holds the ultimate authority in the clinical act, especially as AI moves toward complex in-silico patient modeling [26, 27].

## A systematic approach to implementation

Building calibrated trust requires a systematic, evidence-based approach. As with new medical procedures, physician confidence in AI systems develops through consistent exposure and reliable performance in clinical contexts. The key lies in starting with low-risk applications where AI can demonstrate its value without compromising patient safety, gradually building clinician confidence as familiarity increases [28]. The research demonstrates that trust optimization can be anchored through evidence-based calibration: clinicians verify AI outputs via performance metrics, peer-reviewed validation studies, and cited literature, thereby improving alignment between trust levels and actual utility [29].

This methodical approach prevents both the dangerous extremes of blind reliance and premature dismissal, fostering a mature relationship with AI technology that prioritizes patient safety while embracing innovation. The challenge extends beyond individual clinician attitudes to encompass institutional and regulatory frameworks. Healthcare organizations must establish governance structures that ensure AI tools meet clinical standards while providing the transparency and accountability mechanisms that clinicians require to make informed decisions [26]. This is particularly critical in high-stakes environments like intensive care, where expert consensus is now defining specific integration priorities [30]. Finally, as the UK National Institute for Health and Care Excellence (NICE) stresses in its position paper, the use of AI in evidence generation requires appropriate testing and outcome-blinded assessment to ensure that potential benefits are validated by rigorous clinical standards [22].

## LLM integration and adoption in clinical practice

Integrating LLMs into daily practice does not need to be overwhelming; a gradual, stepwise approach is often more effective than attempting wholesale transformation from the outset. Research shows that doctors who successfully integrate LLMs into their work take a step-by-step approach: they start with simple tasks, learn what works well, and then slowly expand to more complex applications [31]. When hospitals rush to implement LLM-powered documentation tools, they need careful quality checks to make sure these systems are accurate, safe, and that doctors actually want to use them [32].

The practical applications of LLMs in daily clinical practice are both diverse and immediately implementable. At the level of documentation, these tools excel at transforming lengthy clinical notes into concise discharge summaries, drafting structured reports from unorganized consultation notes, and generating preliminary SOAP notes from dictated patient encounters [33, 34]. For patient communication, LLMs can synthesize complex clinical guidelines into patient-friendly educational materials, enabling physicians to provide clearer explanations of treatment plans and procedures [35]. An oncologist, for instance, can input a detailed chemotherapy protocol and receive a simplified, compassionate explanation suitable for direct patient discussion [36]. Similarly, in chronic disease management, new frameworks are demonstrating how AI-assisted pathways can facilitate person-centered care for conditions like asthma and allergic rhinitis [37]. For administrative efficiency, LLMs can create referral letters from brief clinical summaries or draft prior authorization requests from relevant clinical information [33]. The technology also proves particularly valuable in literature review and evidence synthesis, where inputting a structured clinical question yields organized summaries of recent research, complete with strength-of-evidence assessments and clinical implications.

Another emerging development is the use of natural-language prompting to build simple digital tools without traditional programming skills, a practice sometimes referred to in popular literature as ‘vibe coding’ [38, 39]. This may allow clinicians and teams to prototype personalized workflow aids on a small scale, although such tools still require careful validation before clinical use.

What makes some doctors embrace LLMs while others resist them? Research shows it’s not just about the technology itself. Success depends on workplace culture, whether hospital leadership supports the change, and how well LLMs fit into existing routines. Most importantly, doctors are more willing to use LLMs when they see them as helpful assistants rather than replacements for their medical judgment. This reinforces why it’s crucial to present LLMs as collaborative partners in medical decision making rather than threats to physician expertise [40].

## A practical framework for safe integration

Successful LLM integration depends not only on the technology itself, but on workplace culture, institutional leadership support, and how well these tools fit into existing clinical routines [8, 15]. Research consistently shows that physicians are more willing to adopt LLMs when they are positioned as collaborative assistants rather than replacements for clinical judgment [40]. Importantly, integration does not have to be overwhelming: doctors who successfully incorporate LLMs into their practice typically begin with low-complexity tasks, consolidate their experience, and gradually expand to more demanding applications [31]. When institutions rush to deploy LLM-powered documentation tools without adequate quality assurance, adoption fails, underscoring that organizational readiness is as important as technological capability [32].

To provide a structured approach for practitioners, we developed a practical framework for safe LLM use in clinical practice (Table 2). The framework begins with a mandatory Data Protection Check, prohibiting the entry of identifiable patient data into non-compliant systems. It then outlines Risk Awareness, distinguishing lower-risk non-clinical uses such as documentation support or patient education from higher-risk clinical applications requiring strict safeguards and human oversight. It further emphasizes reliance on institutionally approved models and the activation of safeguards such as retrieval-augmented generation (RAG) and citation modes to anchor outputs in trusted evidence.

**Table 2:** Practical framework for using LLMs in clinical practice

Step	Action	Key principle
1. Data Protection	Never input identifiable patient data unless system is secure, compliant and approved by your institution	Privacy first, always
2. Risk Awareness	Distinguish non-clinical (low risk) vs clinical (high risk) uses	Higher risk → stricter safeguards
3. Choosing the Model	Use institution-approved models. Activate safeguards (RAG, citations, uncertainty statements)	Ground outputs in evidence
4. TRUST Safety Loop	Target (clear question) → Reference-check (guidelines, literature) → Understand (plausibility, context) → Save (model, prompt, validation) → Trigger (escalation or termination if unsafe)	Clinician remains final decision-maker
5. Safe Use Zones	Green: patient education, discharge-summary drafting, coding support, literature summarization Amber: differentials, risk explanations (must be checked) Red: autonomous diagnosis/therapy, device programming, triage without oversight	Traffic-light system: safe/cautious/prohibited
6. Monitoring	Re-validate after model updates, track errors, report concerns	Continuous oversight

*LLM* large language model, *RAG* retrieval-augmented generation

**Fig. 1:**
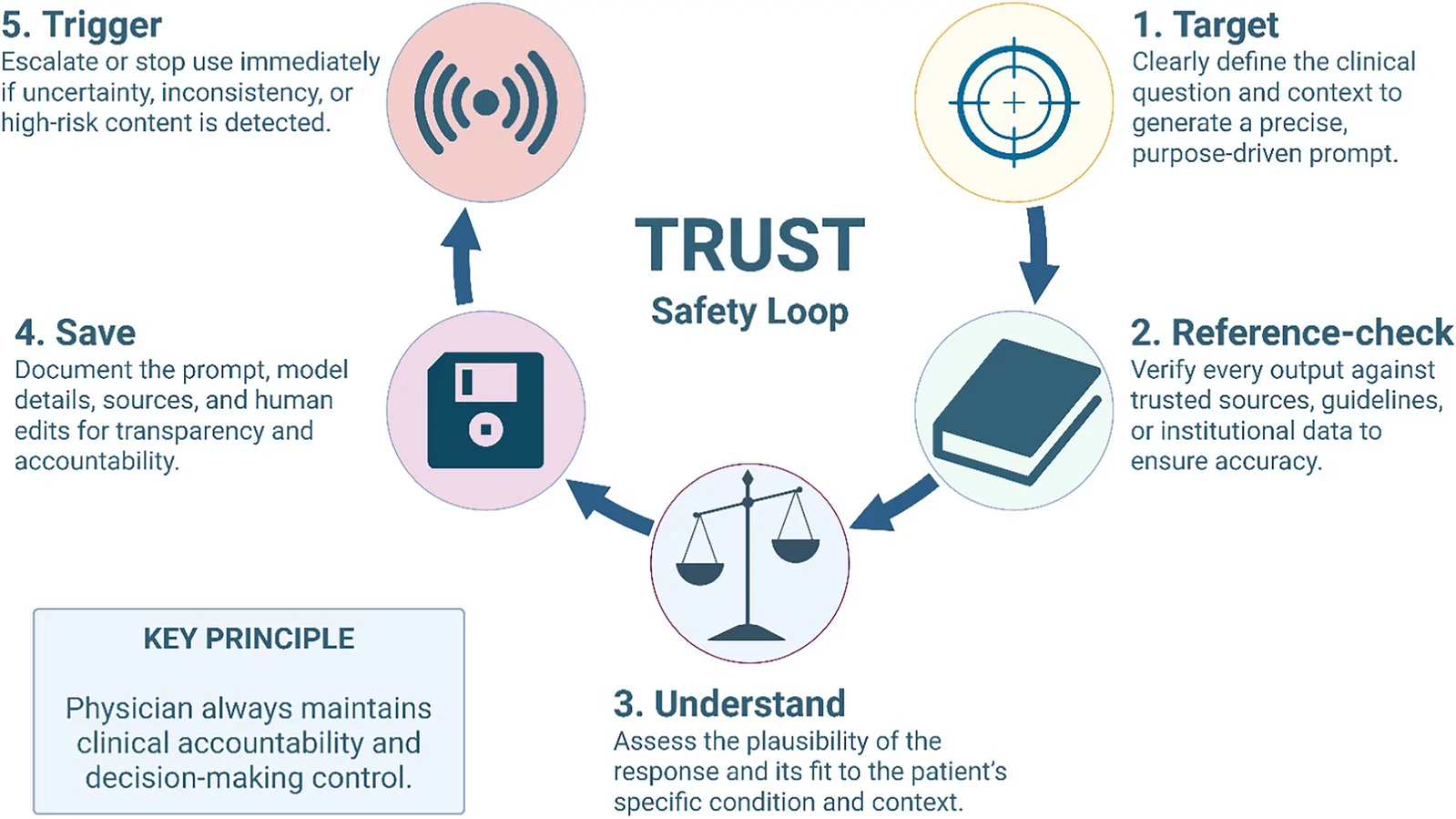
The TRUST safety loop: a structured framework for responsible large language models (LLM) use in clinical practice. Created in BioRender

**Fig. 2:**
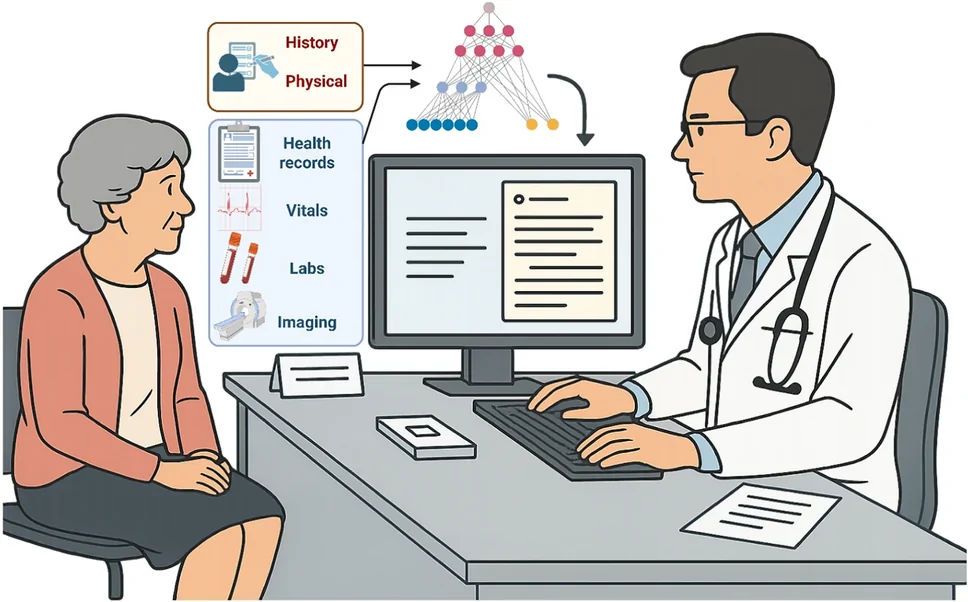
Case vignette: large language model (LLM)-assisted outpatient cardiology visit. A 72-year-old woman with hypertension and chronic coronary syndrome attends a scheduled cardiology follow-up. The electronic health record contains previous discharge letters, ECG and echocardiography reports, and today’s laboratory results. Traditionally, integrating this information into a coherent outpatient letter requires significant time and effort. In this visit, the cardiologist uses an institution-approved large language model (LLM). After entering a short prompt summarizing the encounter (“stable, asymptomatic, well-controlled blood pressure, normal labs”), the model produces a structured draft. The draft incorporates past diagnoses, today’s examination, test results, and laboratory findings into a professional format addressed to the general practitioner. It also suggests a plan section with therapeutic considerations and follow-up. The cardiologist verifies accuracy, adapts content, and documents use of the tool within the TRUST safety framework. Within minutes, a structured draft is generated for physician review, improving efficiency and preserving physician accountability while enhancing time available for patient interaction. Created in BioRender

At its core lies the TRUST Safety Loop (Fig. 1): a mnemonic guiding clinicians to express questions clearly (Target), verify outputs against sources (Reference-check), assess plausibility and patient context (Understand), document the process (Save), and escalate or exclude results if there is any doubt (Trigger). The term TRUST was chosen deliberately, reflecting both the procedural steps and the framework’s overarching purpose: to build trustworthy, traceable, and safe LLM use that supports, rather than replaces, clinical judgment. Applications are further categorized through a traffic-light system defining Safe Use Zones: green for low-risk uses, amber for applications requiring caution and verification, and red for uses not permitted unless the LLM is a certified medical device. A real-world example of this structured workflow is illustrated in Fig. 2.

## Structural and technical constraints: the boundaries of responsible use

### Technical reliability and hallucinations

A basic familiarity with the machine learning architectures powering LLMs is necessary to appreciate their intrinsic limitations, knowledge that cannot be assumed even among frequent users [29]. A fundamental boundary of current technology is the scaling law frontier: the point at which increasing computational power and training data yield progressively diminishing returns in performance, suggesting a practical ceiling that current model architectures have yet to overcome [41, 42].

The most significant clinical risk remains hallucinations, i.e., plausible but fabricated information that in clinical contexts could have serious consequences for patient safety [9, 43]. When Asgari et al. evaluated a safety framework across 450 clinical consultations, they identified a hallucination rate of 1.47% and an omission rate of 3.45% [9]. While most errors were minor, the potential for catastrophic misinformation necessitates constant vigilance. Furthermore, clinicians must distinguish between a model’s static training data and real-time retrieval: unless specifically equipped with real-time search capabilities such as Retrieval-Augmented Generation (RAG), a model’s outputs remain limited by its knowledge cutoff date, potentially missing recently updated clinical protocols or breakthrough research. This underscores the need for domain-specific fine-tuning and systematic bias auditing to prevent the perpetuation of inequities across demographic and disease subgroups [6, 44].

### Systemic realities: economics and environment

The transition from novelty to necessity is governed by significant systemic hurdles. Currently, the long-term cost-effectiveness of LLM integration remains largely theoretical; while these systems promise administrative efficiency, such gains must be balanced against the substantial capital expenditure required for specialized infrastructure, robust data governance, and continuous institutional training [45, 46].

These economic considerations are increasingly inseparable from the issue of environmental sustainability. Although per-query estimates vary and remain method-dependent, the broader systems-level concern is clearer, as the IEA projected doubling of data-center electricity consumption by 2030 in its base case [47]. For healthcare systems, this is not an abstract ecological concern, but a direct threat to public health. The 2024 Lancet Countdown report highlights a record-breaking 167% increase in heat-related mortality among older adults compared to the 1990s, alongside the climate-driven expansion of vector-borne diseases and food insecurity [48].

Healthcare institutions therefore face a profound paradox: adopting AI to enhance clinical efficiency may inadvertently exacerbate the environmental stressors that drive the very diseases these systems are intended to manage. A truly "necessary" AI framework must, therefore, account for its own carbon footprint as a determinant of population health.

### Privacy, security, and regulation

Beyond efficiency and knowledge management, security and ethical considerations remain paramount in LLM healthcare implementation. Khan et al. highlighted how emerging AI innovations in healthcare must balance technological advancement with robust security protocols and ethical frameworks. Successful LLM integration requires not only functional capabilities but also transparent governance structures that protect patient data while maintaining clinical utility [49]. A critical security and governance concern is how LLMs handle protected health information. The Health Insurance Portability and Accountability Act (HIPAA) in the U.S. is considered by some to be outdated and inadequate to address AI-related privacy concerns, as it was created before today’s internet-scale data ecosystem and long before generative AI. This is particularly relevant when physicians include protected patient data in the prompts they submit to LLMs, which would create a privacy problem. The concept of de-identification, which is central to HIPAA, may be a fallacy in the age of LLMs, as data can easily be re-identified. For this reason, it is recommended that physicians and patients alike use these products cautiously, as a lack of privacy erodes trust and promotes exploitation [50]. Similarly, the EU’s General Data Protection Regulation (GDPR) permits access to personal data, including health data, only when there are specific justifications. OpenAI’s ChatGPT has faced investigation and enforcement actions in Europe regarding GDPR compliance [51, 52]. The challenge lies in creating regulatory frameworks that can keep pace with rapidly evolving AI technologies while ensuring patient safety and data protection. This requires unprecedented collaboration between technologists, clinicians, regulators, and ethicists to establish standards that promote innovation while safeguarding the fundamental principles of medical practice.

## Conclusions

Physicians who embrace LLMs as useful collaborators in their practice, while always maintaining firm control over clinical responsibility and decision making, may gain advantages in efficiency, accuracy, and patient care. However, this technological partnership must be built on the foundation of unwavering physician accountability and regulatory oversight. AI may process information, suggest insights, and streamline workflows, but the final clinical decisions, therapeutic relationships, and the moral responsibility for patient outcomes must always remain with the healthcare professional. Mastery takes effort, but the rewards are profound. Rather than walking while a car sits idle, we must learn to drive AI with both hands on the wheel and eyes on the road.

## Data Availability

No datasets were generated or analyzed for this study.

## Abbreviations

ACP: American College of Physicians
AI: Artificial Intelligence
AMA: American Medical Association
ECG: Electrocardiogram
EU: European Union
GDPR: General Data Protection Regulation
HIPAA: Health Insurance Portability and Accountability Act
HTA: Health Technology Assessment
IEA: International Energy Agency
LLM(s): Large Language Model(s)
NICE: National Institute for Health and Care Excellence
RAG: Retrieval-Augmented Generation
SIMI: Italian Society of Internal Medicine
SOAP: Subjective, objective, assessment, plan
